# Information about variations in multiple copies of bacterial 16S rRNA genes may aid in species identification

**DOI:** 10.1371/journal.pone.0212090

**Published:** 2019-02-15

**Authors:** Jerald Conrad Ibal, Huy Quang Pham, Chang Eon Park, Jae-Ho Shin

**Affiliations:** School of Applied Biosciences, Kyungpook National University, Daegu, Republic of Korea; National Cancer Institute, UNITED STATES

## Abstract

Variable region analysis of 16S rRNA gene sequences is the most common tool in bacterial taxonomic studies. Although used for distinguishing bacterial species, its use remains limited due to the presence of variable copy numbers with sequence variation in the genomes. In this study, 16S rRNA gene sequences, obtained from completely assembled whole genome and Sanger electrophoresis sequencing of cloned PCR products from *Serratia fonticola* GS2, were compared. Sanger sequencing produced a combination of sequences from multiple copies of 16S rRNA genes. To determine whether the variant copies of 16S rRNA genes affected Sanger sequencing, two ratios (5:5 and 8:2) with different concentrations of cloned 16S rRNA genes were used; it was observed that the greater the number of copies with similar sequences the higher its chance of amplification. Effect of multiple copies for taxonomic classification of 16S rRNA gene sequences was investigated using the strain GS2 as a model. 16S rRNA copies with the maximum variation had 99.42% minimum pairwise similarity and this did not have an effect on species identification. Thus, PCR products from genomes containing variable 16S rRNA gene copies can provide sufficient information for species identification except from species which have high similarity of sequences in their 16S rRNA gene copies like the case of *Bacillus thuringiensis* and *Bacillus cereus*. *In silico* analysis of 1,616 bacterial genomes from long-read sequencing was also done. The average minimum pairwise similarity for each phylum was reported with their average genome size and average “unique copies” of 16S rRNA genes and we found that the phyla Proteobacteria and Firmicutes showed the highest amount of variation in their copies of their 16S rRNA genes. Overall, our results shed light on how the variations in the multiple copies of the 16S rRNA genes of bacteria can aid in appropriate species identification.

## Introduction

Over the past few decades, the 16S rRNA gene has been generally accepted as a standard for identification and classification of prokaryotic species owing to its structure, containing conserved and variable regions, and occurrence in all organisms. Moreover, its relatively short length allows its easy sequencing [1]. On the contrary, with the increasing availability of sequence information, the limited resolving power of 16S rRNA gene sequences has become obvious, especially when closely related organisms are being inspected [2]. Since the 16S rRNA gene has been used as a standard for classification, presence of multiple copies with sequence variations may pose an obstacle in the classification [3].

In the earlier years, scientists used Sanger sequencing through polyacrylamide plate or capillary gel electrophoresis, both of which were time consuming and laborious [4]. With the introduction of a number of novel and affordable next-generation sequencing technologies, the cost of genome sequencing has decreased rapidly and the clone-based Sanger sequencing of 16S rRNA gene has largely been replaced by various platforms such as 454/Roche (454 Life Sciences, Branford, CT), Illumina (Illumina, Inc., San Diego, CA), and Ion Torrent platforms (Ion Torrent Systems, Inc., Gilford, NH) [5–7].

With technological advancements and emergence of long-read sequencing platforms, such as Single Molecule Real-Time Sequencing (SMRT) by Pacific Biosciences (PacBio) (Pacific Biosciences of California, Inc., Menlo Park, CA), scientists have new opportunities to identify the whole genome sequence (WGS) of an organism, which is a prerequisite in understanding its complete biology. SMRT technology provides an essentially new data type, which has the potential to overcome limitations of the current next generation sequencing platforms, owing to the significantly longer reads, single molecule sequencing, low composition bias, and an error profile compared to other platforms [8]. In the latest PacBio platform, half of the reads are > 14,000-base-pair long and each SMRT cell yields an average of 55,000 reads for the RSII system and 365,000 reads for the Sequel system [9]. PacBio sequencing is a real-time sequencing method and does not require a pause between read steps [10].

Previous studies made use of the entire 16S rRNA sequences, among different genomes, including multiple copy numbers with their variations [1,11–13]. Vetrovský and Baldrian [13] provided an extensive study on the 16S rRNA genes using 1,690 bacterial genomes that entailed the variations present in the multiple copies of 16S rRNA gene. However, although the study used genomes assembled from short-read high-throughput sequencing, the genomes might have an incorrect overlap made between two sequences that both terminate within a repeat element. Moreover, 1.5-kb 16S rRNA gene sequences obtained by the assembly of short reads may have chimera copies from the original multiple copies with irrefutable variations, and often, a minor variable region in a certain copy may be neglected [14] [15]. The genome assembly with SMRT-based long-read sequences is not considered to have many chimeric copies due to incorrect overlaps, since the long-read sequence itself could have acted as a clone contig in the past generation.

Through this advanced and improved technology in sequencing, scientists are now able to determine the exact copy number and variations of 16S rRNA genes present in an organism, and how it impacts the identification process in comparison with the Sanger sequences and 16S rRNA gene sequences available in databases such as NCBI BLAST (https://blast.ncbi.nlm.nih.gov/Blast.cgi) and EzTaxon (http://www.ezbiocloud.net/).

Based on a previous study, the strain *Serratia fonticola* GS2 was observed to have variations in multiple copies of its 16S rRNA gene [16]. In this study, whole genome sequence of a bacterial strain *Serratia fonticola* GS2, which solely used SMRT technology for sequencing, were analyzed. PCR products of 16S rRNA genes from GS2 containing multiple copies with variations were sequenced by traditional capillary gel electrophoresis Sanger sequencing method, and its impact on species identification were addressed. As a result, a comprehensive analysis of the 16S rRNA genes was done by comparing its Sanger sequence with its complete whole genome sequence. We confirmed that the Sanger sequence of PCR products of the 16S rRNA gene existed as chimera or a combination of the copies of the 16S rRNA genes present in the whole genome, and investigated how the chimeric sequence affected classification. Moreover, we also investigated highly similar multiple copies of 16S rRNA gene sequences of different strains of *E*. *coli* and species of *Shigella*, as well as *Bacillus thuringiensis* and *Bacillus cereus*. We also traced all the long-read sequence-based bacterial genomes available at present, examined their 16S rRNA gene copy and variation, and suggested the maximum variation in the resultant chimera PCR product.

## Materials and methods

### Whole genome sequencing data

The complete genome sequence of *S*. *fonticola* GS2, used in this study, was downloaded from National Center for Biotechnology Information (https://www.ncbi.nlm.nih.gov/nuccore) with NZ_CP013913 as its accession number. The 16S rRNA of the whole genome, along with its copies, were analyzed using CLC Genomics Workbench version 9 (CLC Bio, QIAGEN Company, Aarhus, Denmark) and MEGA software version 7 [17].

### 16S rRNA gene cloning of *S*. *fonticola* GS2

Genomic DNA of *S*. *fonticola* GS2 was extracted using Wizard Genomic DNA Purification kit (Promega, Madison, WI), as per the manufacturer’s instruction. The product was then subjected to PCR using the 16S rRNA universal primers 27F and 1492R. Initial denaturation was at 95°C for 3 min, followed by 30 cycles of denaturation at 95°C for 20 s, annealing at 57°C for 30 s, and extension at 72°C for 90 s, and a final extension at 72°C for 5 min. The PCR product was purified using EZ-Pure PCR Purification Kit ver.2 (Enzynomics, ROK). Cloning was done using pTOP Blunt V2 vector TOPcloner Blunt Kit (Enzynomics, ROK) according to the manufacturer’s instructions. The product was transformed into *E*. *coli* DH5α competent cells via heat shock method. The transformed cells were spread on LB agar containing ampicillin.

### Plasmid extraction and sequencing

Sixteen colonies were randomly picked and used for plasmid extraction. Plasmids were extracted using GeneJET Plasmid Miniprep Kit (Thermo Fisher Scientific, MA), following manufacturer’s instructions. Only 6, out of the 16 colonies picked, were deemed suitable for sequencing and labeled as Clones A, B, C, D, E, and F, respectively. The products were checked if there are presence of other sequences through gel electrophoresis. The appearance of only one band size of about 1500 kb suggests that there were no artifacts presence that could interfere with sequencing. The plasmids were sent to Macrogen (Daejeon, Korea (http://www.macrogen.com) for sequencing, using the primers M13 (-20F) and M13 (-48R); the sequences obtained were analyzed using MEGA software version 7. The sequences were uploaded in NCBI and have the following accession numbers. MK235154, MK235155, MK235156, MK235159, MK235160, MK235161, and MK235162 for Clones A, B, C, D, E, F, and the Actual PCR sequence, respectively.

### *In-silico* analysis of long-read sequencing of bacterial genomes

First, we downloaded 22,340 publicly available sequences from long-read sequencing machine (searched by keywords as "Sequencing Technology :: PacBio"; "Sequencing Technology :: PacBio RS"; "Sequencing Technology :: PacBio RS II"; "Sequencing Technology :: PacBio RSII"; "Sequencing Technology :: Pacific Biosciences"; "Sequencing Technology :: Pacific Biosciences RS"; "Sequencing Technology :: Pacific Biosciences RS II"; "Sequencing Technology :: Pacific Biosciences RSII"; "Sequencing Technology :: SMRT”, "Sequencing Technology :: PacBio Sequel", "Sequencing Technology :: Oxford Nanopore", "Sequencing Technology :: MinION", "Sequencing Technology :: Oxford Nanopore Technologies") on INSDC GenBank database (http://www.ncbi.nlm.nih.gov/genbank/) version 2018-08-20. An in-house Python script detailed in the Supporting information (S1 File) was used to extract complete genomes (with accession numbers having “CM”; “AC”; and “CP” prefix, but not as contig or plasmid) along with information about their 16S rRNA sequences and taxonomy data from raw GenBank file. In order to examine the similarity of 16S rRNA copies in the genome of a species, the 16S rRNA sequences were multiple aligned by Clustal Omega software with default parameters (http://www.clustal.org/omega/). Afterwards, for grouping 16S rRNA genes into “unique copies”, we used SNP-sites program (https://github.com/sanger-pathogens/snp-sites) to export a list of variants and classified 16S rRNA copies based on variant positions to define “unique copies”. Additionally, the genomes with nonexistent 16S rRNA gene copies in their annotation, or which contain INDELs (insertion-deletion) within 16S rRNA gene copy alignment file were not included in the analysis.

## Results and discussion

### Sanger sequencing and characterization of *S*. *fonticola* GS2 16S rRNA gene copies

**Fig 1:**
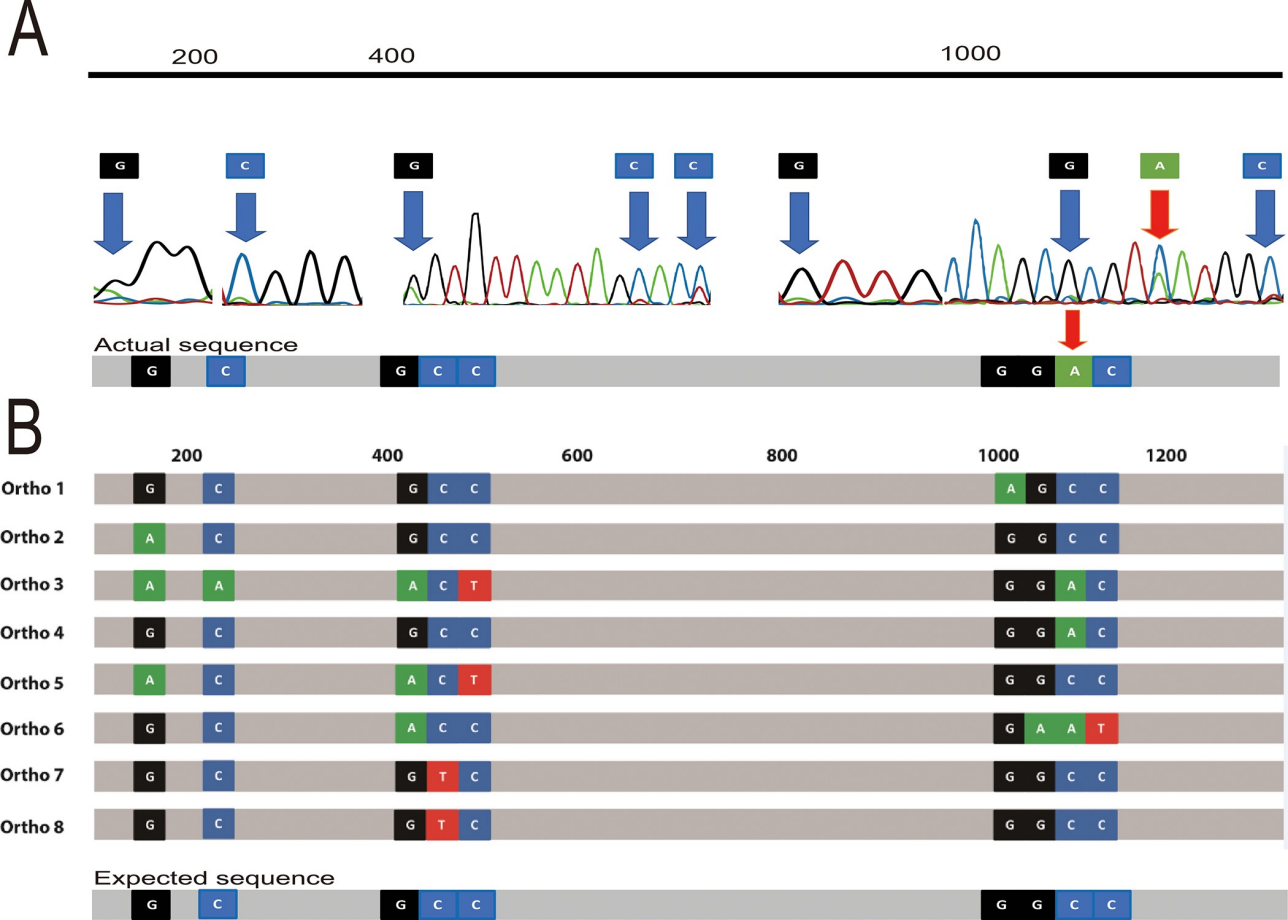
Double peaks from Sanger sequencing electropherogram of the 16S rRNA-gene PCR product of strain GS2. (A) shows the actual PCR product sequence and location of variants in the electropherogram, where double peaks are found, and (B) shows the actual sequence vs. expected sequence of the variants where the actual sequence is the sequence obtained from Sanger sequencing and the expected sequence is the sequence derived from the alignment of the 8 copies of the 16S rRNA genes of GS2. The red arrow indicates the base where sequencing machine showed a deviation from the 16S rRNA sequence of GS2. Nucleotides were color-coded based on the electropherogram result; G (black), T (red), A (green), and C (blue) and nearest locations where the variations are found in the 16S rRNA genes are placed above the electropherogram.

Using the PacBio RSII system, the whole genome of *S*. *fonticola* GS2 was sequenced and was found to have multiple copies of its 16S rRNA genes [16]. Investigation of the multiple copies showed variation among them. This part of the study was aimed to investigate the difference between the 16S rRNA genes from Sanger sequencing of PCR products and the 16S rRNA genes from whole genome sequencing. Before Sanger sequencing, the PCR product of 16S rRNA gene was purified and its good quality was ensured as described in the materials and methods. Initial Sanger sequencing for the identification of *S*. *fonticola* GS2 showed multiple double peaks in the electropherogram (Fig 1), which suggested that there are other nucleotides in the sample. In the electropherogram, although there was a higher peak for base C, the sequencing machine, owing to its algorithm, put A instead (Fig 1A and 1B, position of red arrow). These deviations may have originated from laboratory procedures and the sequencing technique used [18]. Another possible reason for multiple double peaks may be the occurrence of variation or heterogeneity in the 16S rRNA copies present in the genome of interest. A study by Reischl et al, showed multiple peaks in the electropherogram of *Mycobacterium celatum* due to the presence of two different copies of 16S rRNA genes [19]. In another study, visual inspection of the electopherograms also showed double peaks in the genus *Neisseria*, and the authors suggest this heterogeneity in sequencing signals probably represents real base differences among different 16S rRNA gene sequences [20]. These sequence variations in copies of the 16S rRNA gene may be explained by multiple phenomena. According to Acinas et al [11], a functional rRNA gene, produced from horizontal gene transfer, would display nucleotide alterations that are concentrated in variable regions and compensated for, if present in the stem regions. Moreover, variation of the copies may arise from mutations and, therefore, classified by Sun et al [21] into five categories: intervening sequence (IVS), inserts larger than 10 nucleotides, deletion, truncation, and regional diversity or random.

We hypothesize that PCR products may form a variety of chimera from the 9 variant points, but the most frequently occurring base would possibly be selected in the final sequence, due to the characteristics of Sanger sequencing method that simultaneously detect the luminescence of millions of molecules (Fig 1B, Expected sequence). To determine which double peaks are of interest, we based it on sequences of the 8 copies of the 16S rRNA genes of GS2 from the whole genome sequence. We first aligned the 8 copies of the 16S rRNA genes of GS2. In doing so, we were able to determine the locations with variations. In our analysis, we have found 9 locations in the 16S rRNA gene of GS2 which vary from each other. Using the same method, we searched for the sequences positioned before and after the variation site, we were able to pinpoint the location where the double peak would exist. In the *S*. *fonticola* GS2 genome, by aligning the 8 copies of the 16S rRNA genes from WGS, we found 9 variant points (Fig 1A, blue arrows). In Fig 1B, the actual product which is the product from Sanger sequencing showed high similarity with the expected sequence, which is the sequence that is deduced from WGS based on the frequency of the nucleotides present in the variation location, except for a base.

### Isolation of mono-clones of 16S rRNA-gene PCR product

**Fig 2:**
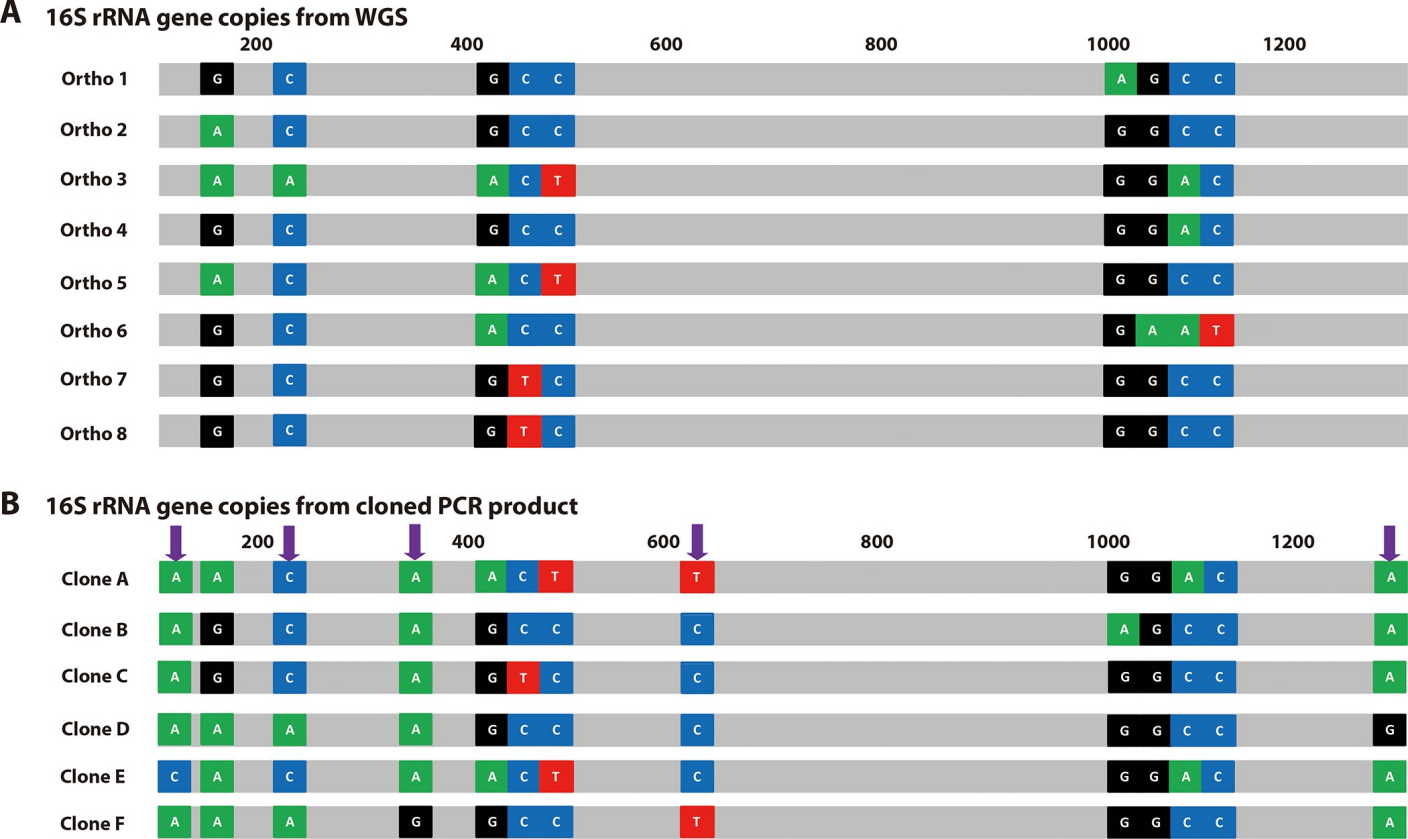
Representation of the number of 16S rRNA copies in GS2. Whole genome sequencing data is shown in (A) (Ortho 1–Ortho 8), and cloned 16S rRNA genes (Clone A–Clone F) with their variations are shown in (B). Nucleotides were colored based on their color-coded based from the electropherogram result; G (black), T (red), A (green), and C (blue) and nearest locations where the variations are found in the 16S rRNA genes are placed above the schematic. The purple arrows show where the region of the *Taq* polymerase error produced variations.

As described above, direct sequencing of the PCR product resulted in a combination of chimeric strands. To determine the variation in each strand, the PCR-amplified strands of 16S rRNA gene were cloned into pTOP blunt V2 vector, and plasmid DNA from 6 different clones was sequenced by Sanger sequencing method. The sequences of PCR clones were compared with the 8 copies of 16S rRNA gene from WGS. The *S*. *fonticola* GS2 genome contained 8 copies of the 16S rRNA gene (Fig 2A, Ortho 1–8). Only Ortho7 and 8 were 100% matched while the other 6 orthologs contained more than one variation. The six PCR clones also had variations in the same regions (Fig 2B). Additionally, four more variant points that did not exist in the WGS orthologs were observed (Fig 2B, blue arrows). This maybe from *Taq* DNA polymerase error during the elongation step of DNA replication. Pairwise similarity and phylogenetic analysis showed that among the six clones, only clone C shares 100% similarity with Ortho 7 and 8 (Fig 2A and 2B). Usually, one would expect that after isolation, a clone sequence having 100% similarity with one of the whole 16S rRNA gene sequences may be readily obtained. However, in this study, five clones out of six did not show any match with the orthologs from WGS. The lowest similarity between the orthologs and clones was found between Ortho 7 and 8 to Clones A and E, and Ortho 6 to Clone F (99.53%) had a maximum difference of 7 base pairs.

Most of the clones showed a combination of sequences based on the 16S rRNA genes from WGS. The intracellular polymorphisms might as well cause difficulties in obtaining an easily interpretable sequence [22]. During PCR, the polymerase would potentially pick up different copies of the 16S rRNA gene and co-amplify it, resulting in a chimeric sequence [23]. A chimeric section is produced when a fragment of DNA from one gene anneals to a homologous template to prime the next cycle of DNA synthesis. The main cause of this chimeric formation is prematurely terminated DNA strands, especially during the later cycles of PCR [24]. Regions of rRNA hairpin structure are commonly encountered that strongly hinder polymerases and may cause premature termination bands across all four sequencing lanes [25, 26].

### Effect of concentration of PCR template

It is a common procedure for scientists to isolate microorganisms and proceed to amplify the DNA via PCR and sequence the 16S rRNA genes for species identification. As stated above, the sequence obtained from PCR product may vary depending on the polymorphism within the microbial genome. Although mainly the dominant base in the PCR product is expected to be read, there may be a sequence base calling error depending on the instrument’s analysis program.

**Fig 3:**
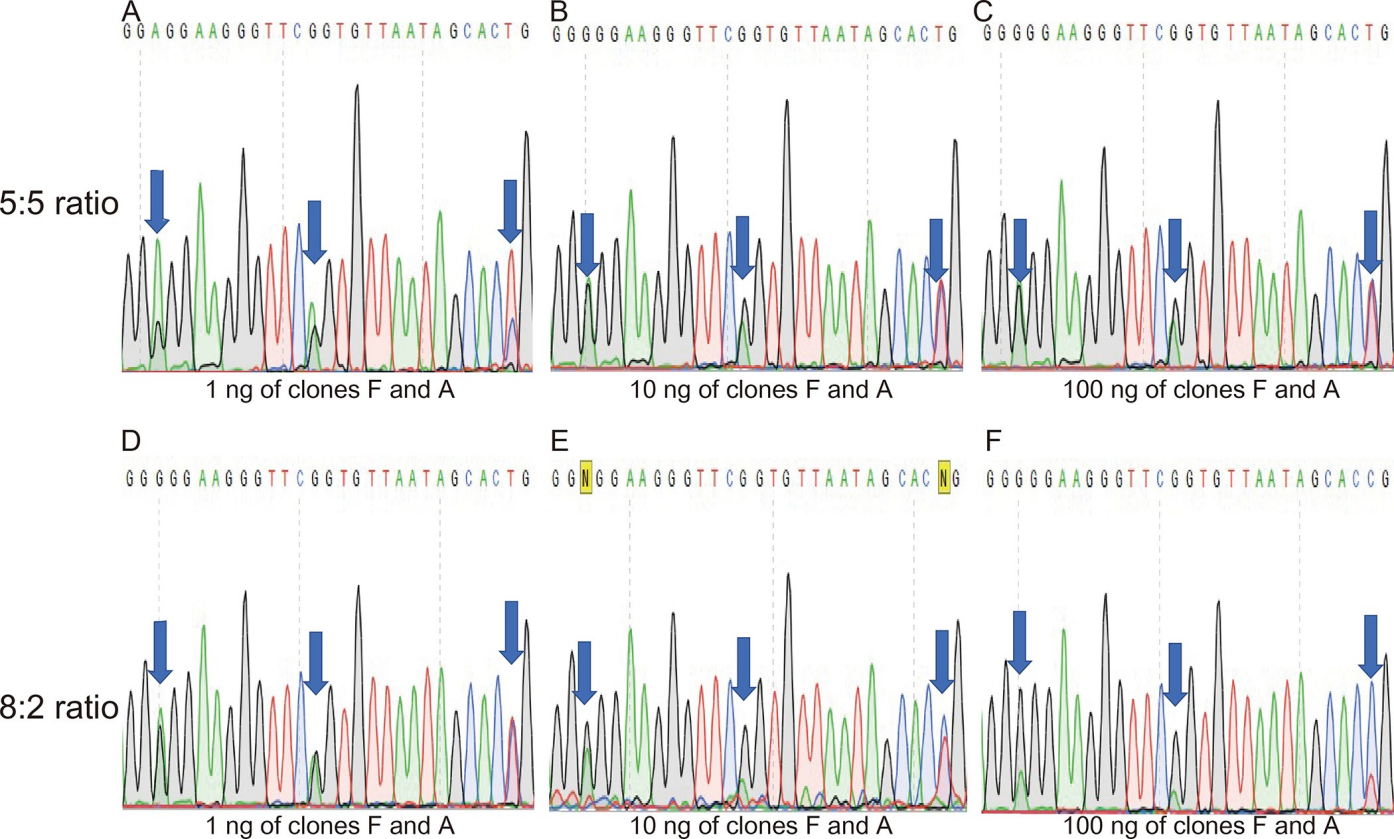
Effect of the amount of PCR template and ratio on the electropherogram and sequencing results. A, B, and C correspond to 5:5 ratio of clones F and A with 1, 10, and 100 ng of PCR template concentration while D, E, and F correspond to 8:2 ratio of clones F and A having 1, 10, and 100 ng of PCR templates, respectively. The electropherograms showed are based from the 400–420 bp region of the 16S rRNA gene Sanger sequencing which showed multiple variations between the Clones F and A.

In addition, different bases may be selectively read according to the absolute amount of PCR template used. In particular, there may be sequence read variations between using the same clone in PCR and normal PCR results, using the same extracted genomic DNA as template. Here, PCR reactions with various templates were designed to address these questions and the results are shown in Fig 3. Plasmids of Clones A and F were chosen as the PCR templates since the clones shared 3 variations within 20-bp windows in the 400–420 bp locations inside the 16S rRNA genes. Clone A provided A-A-T while Clone F showed G-G-C nucleotide variations; both clones showed only single peaks when sequenced (S1 Fig). The clones were first diluted having the same volume and concentration. The ratio of plasmids used as PCR templates was 5:5 and 8:2 for Clones F and A, respectively which were both based on volume of the initial dilution. The 8:2 ratio was chosen randomly to check the effect of the ratio between a high and low concentration while the 5:5 ratio was used for equal ratios, in that way we were able to compare the electropherograms. PCR amplification was performed with three different template amounts (100, 10, and 1 ng of mixed plasmids per 100 μL of PCR reaction).

Having equivalent ratio of 5:5, at low concentration (1 ng, 33 fM, 3.3 M^-16^), the electropherogram showed that the higher peaks belonged to Clone A (A-A-T), although the program read the central A as G (Fig 3A). However, at 10 ng (0.33 pM, 3.3 M^-15^), the higher peaks and corresponding base differed, resulting in a combination from Clone A and Clone F (Fig 3B). The same pattern was observed with 100 ng (3.3 pM, 3.3 M^-14^, Fig 3C). In the 8:2 ratio of Clones F and A, peaks from Clone A were not dominant at low concentration (1 ng, 33 fM, 3.3 M^-16^, Fig 3D). Strong separation of peaks was observed only at a higher concentration of the PCR template (100 ng, 3.3 pM, 3.3 M^-14^, Fig 3F). It is said that the absolute peak height in the electropherogram depends on the amount of template DNA in the sequencing reaction [27]. Having equivalent ratio of 5:5, A showed a higher peak compared to G (Fig 3A) at 1 ng template, whereas at increased template concentrations of 10 and 100 ng, G was the base called, instead of A (although peak separation was not clearly shown). There appears to be preferred bases in Sanger sequencing with a low template concentration. Although we can’t clarify the reason behind this, it is still a good idea to keep the template concentration above the appropriate level for accurate base determination.

At a given location of variant base pairs, due to multiple copies in the 16S rRNA gene, the base pair present in higher amount during sequencing has a greater chance of being amplified and eventually appear in the sequencing result. It is worth noting that even though nucleotide A showed a higher peak, the base that was called while sequencing was G, at 1 ng template concentration (Fig 3D). On the other hand, at the template concentration of 10 ng, N was placed instead, due to unclear separation of peaks (Fig 3E). From two variants and three concentrations of template DNA, four different sequencing reads were obtained. Therefore, it is presumed that a variety of sequences may be obtained by PCR and Sanger sequencing of the actual 16S rRNA gene.

### Effect of multiple copies of 16S rRNA gene in species identification

**Fig 4:**
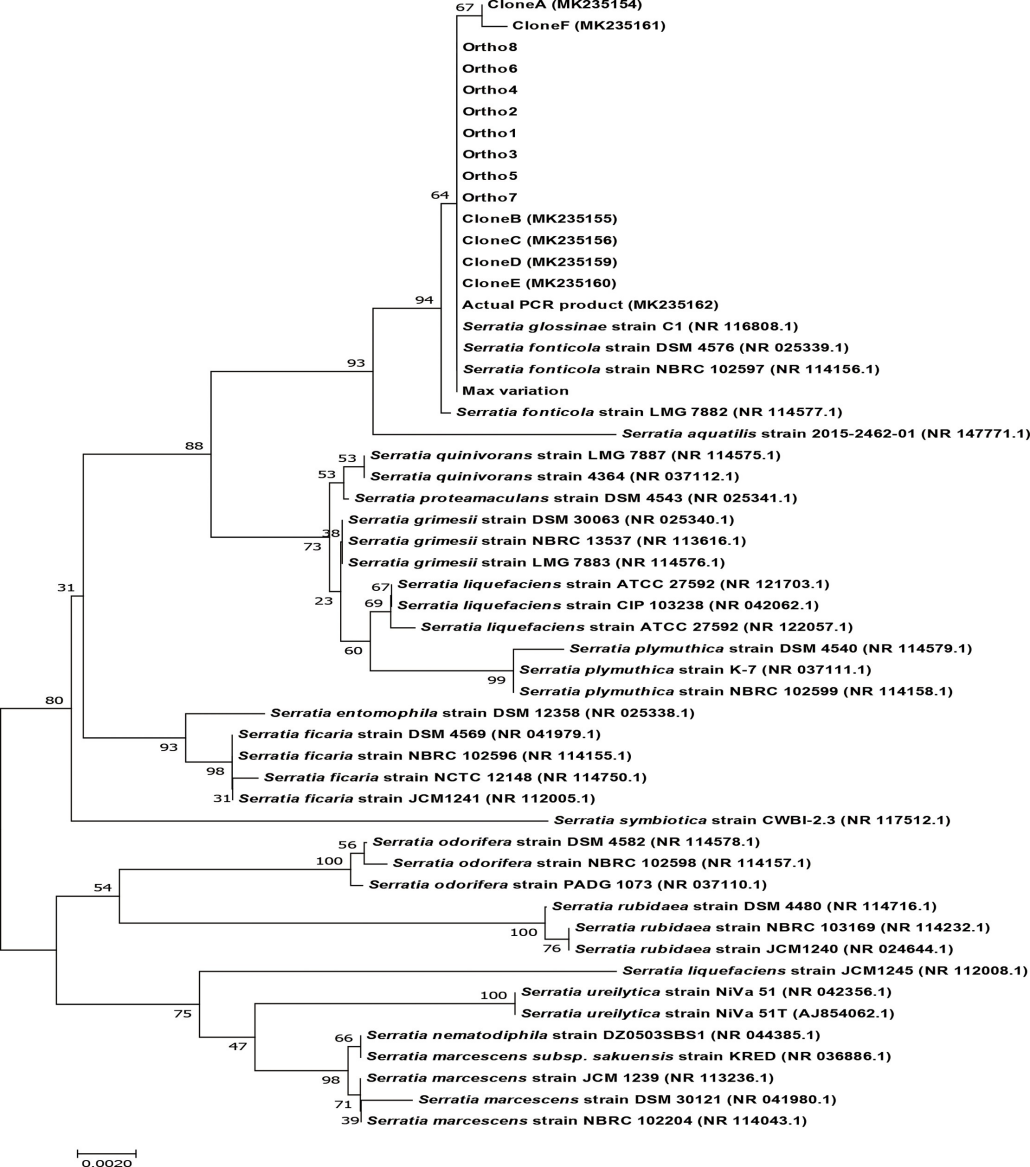
Phylogenetic tree of 16S rRNA genes from WGS data of *S*. *fonticola* GS2 with the cloned PCR products, actual PCR product, and the sequence that mimics the maximum variation. Copies of the 16S rRNA genes from WGS (Ortho1-8), cloned PCR products (Clone A-F), actual PCR product, and a mimic of the sequence with the highest variation, were compared with other species from the genus *Serratia* using a phylogenetic tree. Bootstrap values obtained with 1000 repetitions are indicated as percentages at all branches. The scale bar represents an evolutionary distance of 0.0020. GenBank accession numbers of the different *Serratia* species are provided in parentheses.

To check whether the chimeric structures of 16S rRNA gene have an effect on species identification, a phylogenetic tree was constructed. Using the data from WGS, they were compared with different *Serratia* species, found in NCBI, with at least 1400 bp in their sequence to avoid bias in the construction of the tree (Fig 4). The phylogenetic tree generated showed that there was no change in species identification of GS2 for all the copies of 16S rRNA genes, including the sequence that imitates one with maximum variation. Inclusion of 8 copies of 16S rRNA genes found in WGS, 6 cloned PCR products, the actual PCR product, and a sequence in which the base N was used in 9 variation points observed in Fig 1A (maximum variation) were closely related to *Serratia fonticola*.

Despite a drop in percentage similarity among the sequences, all the sequences that changed remained closely similar to that of *S*. *fonticola* LMG 7882 and *Serratia glossinae* C1, which is a later synonym of *S*. *fonticola* [28]. Although multiple copies and heterogeneity exist among the 16S rRNA genes present in GS2, there was no change in the identity of the species, as observed from the phylogenetic trees. Generally, heterogeneity in the copies of 16S rRNA genes is limited, and unlikely to have an effect on the classification of taxa [1, 29]. A minimum percentage similarity of 99.42% was observed between *S*. *fonticola* GS2 and the sequence with the maximum variation. Assuming that no error occurred during sequencing, if a different strain of *S*. *fonticola*, was compared with GS2, having a lower percentage in comparison to the minimum pairwise similarity, the strain being compared to GS2 would possibly be different. In order to identify whether two species are of the same strain, whole sequence of their 16S rRNA gene, along with their copies, must be studied.

Species identification is a delicate and complex matter, especially species with highly similar sequences. In order to assess if our theory about the maximum variation and minimum similarity are able to distinguish closely related species, we tried to separate *E*. *coli* with *Shigella* and the infamous *Bacillus thuringiensis* and *Bacillus cereus*. For the case of *E*. *coli* and *Shigella* [4] which have a high similarity among their 16S rRNA gene copies, we first used all of the copies of all the 16S rRNA genes that are present in the long-read sequences database to generate a phylogenetic tree and check which of the copies are present in the same clade. Using our theory that the more bases that are present in the location where the variation is present, then the more likely the base will be called and will show up in the sequencing result, we generated 16S rRNA gene sequences that we called as “expected sequence” and created a phylogenetic tree using the available 136 strains of *E*. *coli* and 7 *Shigella* species (Fig not shown).

Based on the generated tree, 110 *E*. *coli* were clearly placed in outgroups with respect to *Shigella*. Therefore, we narrowed down the *E*. *coli* and *Shigella* strains to those that share the most similar sequences of their 16S rRNA genes (26 *E*. *coli* strains and 7 *Shigella* species). Using the 26 *E*. *coli* strains, the expected sequences were compared with the expected sequences for *Shigella*. Aside from one *Shigella* (*Shigella* sp. PAMC 2870) we were able to separate the *E*. *coli* strains from the other *Shigella* species (S2 Fig). It should be noted that the *Shigella* sp. PAMC 2870 doesn’t have a complete identification of its species. A simple schematic (S1 Table) shows the variation points where multiple nucleotides exist in the 16S rRNA gene copies of 7 *E*. *coli* strains and 7 *Shigella* species and their expected sequences.

A famous example of closely related 16S rRNA gene sequences is the case for *Bacillus thuringiensis*, *Bacillus cereus*, and *Bacillus anthracis*. The three Bacillus species are widely known to have different phenotypes however their 16S rRNA genes are very similar. Many have differentiated the three species using their phenotypes and different toxin genes [30,31]. From our data, we found no WGS of *Bacillus anthracis*, however, using the same method described above, we generated the expected sequences for *Bacillus thuringiensis* and *Bacillus cereus*. We found only one variation site in all of the expected sequence for all the strains of *Bacillus thuringiensis* and *Bacillus cereus* that are present in our data. Only *Bacillus thuringiensis* serovar coreansis ST7 shared the single difference in nucleotide with the other strains of *Bacillus cereus*. The expected sequences for both species which have different strains were not separated in the phylogenetic tree (S3 Fig).

Despite the presence of multiple copies with variations, use of 16S rRNA genes for taxonomy is not affected and, therefore, sufficient for most species identification except for a special case involving *Bacillus thuringiensis* and *Bacillus cereus*.

### *In-silico* analysis of the 16S rRNA genes of long-read-based bacterial genomes

With the increasing availability of sequences, an *in silico* analysis of the 16S rRNA genes of bacterial genomes that are based from long-read sequencing that are present in NCBI was done. Similar to what we’ve done with the closely related species, here we report the maximum variation and the minimum similarity of genomes which are currently present in the NCBI database. We theorize that this minimum similarity can be a threshold for species identification. There are approximately 34 known bacterial phyla (http://www.bacterio.net/-classifphyla.html, 2018), out of which, only 19 were identified in the 2235 genomes that used long-read sequencing technology exclusively, including a candidate phylum *Candidatus Dependentiea*. The whole genome sequences, obtained from NCBI, were obtained only using long reads from PacBio and Oxford Nanopore Technologies. Among the 2235 genomes obtained, only 1616 were processed in order to avoid sequences that contained indels which could provide bias in the calculation of the maximum variation for each bacteria. The 16S rRNA gene copy number, genome size, and unique variations from each copy are summarized in Table 1. Genomes that are included in the phylum Firmicutes and Proteobacteria were observed to have the largest gene copy numbers of 6.788 ± 2.694 and 5.141 ± 2.411, respectively. Consequently, both phyla showed the highest number of variations in the 16S rRNA genes, having values of 5.298 ± 7.052 for Proteobacteria and 5.458 ± 5.633 for Firmicutes. As a result of having the maximum variations in their 16S rRNA gene copies, these two phyla had the least minimum similarity with an average of 99.658 ± 0.454% for Proteobacteria in which *Serratia fonticola* belongs to and 99.649 ± 0.362% for Firmicutes.

**Table 1:** Summary of analyses done on whole genome sequences involving 1616 genomes in different phyla that exclusively used pacific biosciences and oxford nanopore technology sequencing platforms.

Phylum	Number of genomes analyzed per phylum^a^	Average Genome Size (Mbp)^b^	Average 16S rRNA gene copy number^c^	Average number of 16S rRNA genes with unique copies^d^	Average maximum variation^e^	Average minimum similarity (%)^f^
**Actinobacteria**	174	4.740±2.422	3.402±1.720	1.569±0.869	1.121±2.400	99.927±0.158
**Armatimonadetes**	2	2.810±0.249	1	1	0	100
**Bacteroidetes**	104	3.961±1.195	3.712±1.562	1.577±0.932	1.788±4.458	99.883±0.292
**Candidatus Dependentiae**	1	1.17	2	1	0	100
**Chlamydiae**	2	1.102±0.060	1	1	0	100
**Chlorobi**	2	2.407±0.012	2	1	0	100
**Chloroflexi**	2	2.167±1.101	1.5±0.707	1.5±0.707	0.5±0.707	99.967±0.0473
**Cyanobacteria**	7	5.351±1.543	3.2856±1.704	1.714±0.951	2.429±4.392	99.837±0.295
**Deinococcus–Thermus**	6	2.7578±0.604	2.667±0.516	1.5 ±0.837	0.667±1.033	99.956±0.068
**Elusimicrobia**	1	1.580	1	1	0	100
**Firmicutes**	496	3.141±1.213	6.788±2.694	4.423±2.698	5.458±5.633	99.649±0.362
**Fusobacteria**	6	2.262±0.103	4.833±0.408	2.167±1.169	1.667±1.862	99.891±0.122
**Nitrospirae**	1	2.709	2	1	0	100
**Planctomycetes**	1	7.50	3	1	0	100
**Proteobacteria**	1374	4.654±1.642	5.141±2.411	3.044±2.382	5.298±7.052	99.658±0.454
**Spirochaetes**	10	2.634±1.595	1.3±0.483	1.1 ±0.316	0.3±0.949	99.980±0.063
**Tenericutes**	37	0.951±0.260	1.324±0.580	1.297±0.571	0.946±2.828	99.938±0.184
**Thermotogae**	4	1.925±0.219	3.5±1	1	0	100
**Verrucomicrobia**	5	3.970±0.737	1.6±0.894	1	0	100

^a^ number of genomes that used PacBio-based sequencing, analyzed in each phylum

^b^ average genome size with standard deviation for each phylum in megabase pairs

^c^ average number of 16S rRNA gene copies of the genomes analyzed in a phylum

^d^ average number of 16S rRNA gene “unique” copies, where “unique copies” mean the 16S rRNA copies with different variations and are not 100% similar with other copies in a genome

^e^ average maximum variation found in each phylum based on the multiple copies of the 16S rRNA gene

^f^ the percentage generated from the maximum variation divided by the total length of the 16S rRNA genes in different phyla

The number of “unique copies” from the long-read-based whole genome sequences was analyzed next. We identified these “unique copies” by locating points where variation exists in the 16S rRNA genes and comparing them with other 16S rRNA gene copies within a genome. These “unique copies” are copies of 16S rRNA gene that does not show a 100% sequence match with the other copies present within the genome. The highest number of average “unique copies” of 16S rRNA genes was 4.423 ± 2.698 for Firmicutes and 2.705 ± 2.642 for Proteobacteria.

Konstantinidis et al [32] suggested that copy number is positively associated with how the organism responds to resource availability. Nemergut et al [33] determined that through succession, the number of rRNA operons of the community decreased. The array of rRNA genes in bacteria provides a genetic indicator of ecological strategy for utilization of nutrients by a bacterial species [34]. A study by Roller et al [35] found that rRNA copy number is a reliable and generalizable proxy for bacterial adaptation to resource availability.

## Conclusions

*S*. *fonticola* GS2 has multiple copies of the 16S rRNA gene as well as variable sequences amongst these copies. To check whether this would have an effect on taxonomy classification, comparison between Sanger sequence and WGS of the 16S rRNA gene of *S*. *fonticola* GS2 was performed. Sequencing showed chimeric copies of the rRNA genes. Effect of PCR template concentration on sequencing was examined. Although PCR seemed to show randomness in sequence amplification, at sites of variation, greater number of copies in the template were seen to be associated with greater chance of amplification, as shown by the 5:5 and 8:2 ratios of Clone F and Clone A. Comparison with other species, based on simulated chimeric sequences of 16S rRNA genes of GS2, was conducted by constructing phylogenetic trees. The minimum similarity of 99.42% generated by the variations present in the copies was sufficient criterion for not classifying GS2 as a different species. This threshold, however, may be used to differentiate between strains. Species among *Shigella* and *E*. *coli* and *Bacillus thuringiensis* and *Bacillus cereus* strains which are known to have highly similar sequences of their 16S rRNA gene copies were also investigated. Using the expected sequence, we were able to separate the *E*. *coli* strains from the *Shigella* species. Results in the present study suggest that although there was heterogeneity among the multiple copies, it did not affect species taxonomy; thus, the use of the 16S rRNA gene is appropriate for species identification at the current stage except in the special case of *Bacillus thuringiensis* and *Bacillus cereus*. In order to confirm that two species are of different strain, the whole sequence of their 16S rRNA gene, along with their copies (if they have multiple copies), must be considered, as observed in this study. An *in-silico* analysis was also performed using 1,616 genomes downloaded from NCBI, which exclusively used long read sequencing. A total of 18 phyla were observed, with Proteobacteria and Firmicutes being the most sequenced with 1,374 and 496 WGS, respectively. These two phyla showed the maximum number of copies and most variations in their 16S rRNA genes. Since the 16S rRNA genes still remain the chosen marker for bacterial identification, we strongly believe that this insight into the variations of multiple copies may aid in proper bacterial species classification.

## Supporting information

S1 Fig Single peaks from Sanger sequencing electropherogram of the 16S rRNA gene cloned PCR product of Clone F and A. **(**A) Blue arrows shows the single peaks of clone F (G-G-C) in the 400–420 bp region and (B) shows the single peaks of clone A (A-A-T).
(TIF)

S2 Fig Phylogenetic tree of 16S rRNA gene expected sequences from WGS data of 26 different strains of *E*. *coli* and 7 *Shigella* species. 16S rRNA gene expected sequences from WGS of *E*. *coli* strains and *Shigella* species which are highly similar were compared using a phylogenetic tree. Bootstrap values obtained with 1000 repetitions are indicated as percentages at all branches. The scale bar represents an evolutionary distance of 0.001. GenBank accession numbers of the different *E*. *coli* strains and *Shigella* species are provided in parentheses.
(TIF)

S3 Fig Phylogenetic tree of 16S rRNA gene expected sequences from WGS data of 7 different strains of *Bacillus cereus* and 4 *Bacillus thuringiensis*. 16S rRNA gene expected sequences from WGS of *Bacillus cereus* and *Bacillus thuringiensis* strains which are highly similar were compared using a phylogenetic tree. Bootstrap values obtained with 1000 repetitions are indicated as percentages at all branches. The scale bar represents an evolutionary distance of 0.00005. GenBank accession numbers of the different *Bacillus cereus* and *Bacillus thuringiensis* strains are provided in parentheses.
(TIF)

S1 Table Representation of the expected sequences of 7 *E*. *coli* strains and 7 *Shigella* species with the amount of different bases in their respective variation points. (DOCX)

S1 File In-house python script for extracting the whole genome sequences and their 16S rRNA gene information. An in-house Python script used to extract complete genomes (with accession numbers having “CM”; “AC”; and “CP” prefix, but not as contig or plasmid) along with information about their 16S rRNA sequences and taxonomy data from raw GenBank file downloaded on 2018-08-20. First, the genomes that don’t have annotations of their 16S rRNA genes were separated. After the extraction of the 16S rRNA genes, the copies were aligned using Clustal Omega software with default parameters. Those with insertions and deletions were again separated and the variations of the copies were checked using SNP-sites program to export variant list file and classified 16s rRNA copies based on variant positions to define whether or not “unique copies”. The maximum variation, which is the total number of variations in the copies of their 16S rRNA genes were divided by the total length of their 16S rRNA genes to calculate for the minimum percent similarity.
(ZIP)
