# Supplementary figures and images for: Information about variations in multiple copies of bacterial 16S rRNA genes may aid in species identification
Source: PLoS One. 2019 Feb 15;14(2):e0212090. doi: 10.1371/journal.pone.0212090 (PMC6377111; doi:10.1371/journal.pone.0212090)

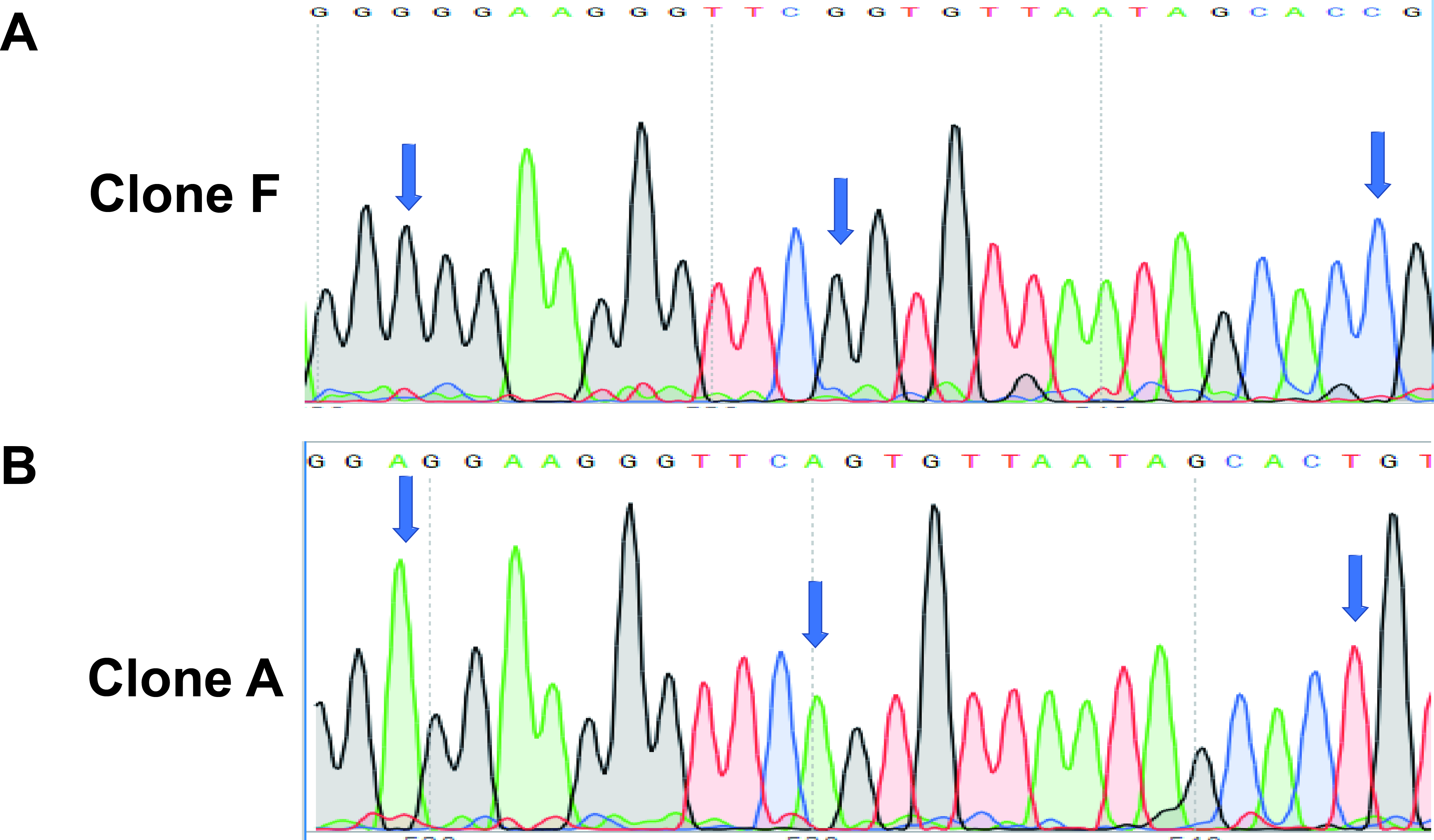

Supplement: S1 Fig — (A) Blue arrows shows the single peaks of clone F (G-G-C) in the 400–420 bp region and (B) shows the single peaks of clone A (A-A-T). (TIF) [file pone.0212090.s001.tif]

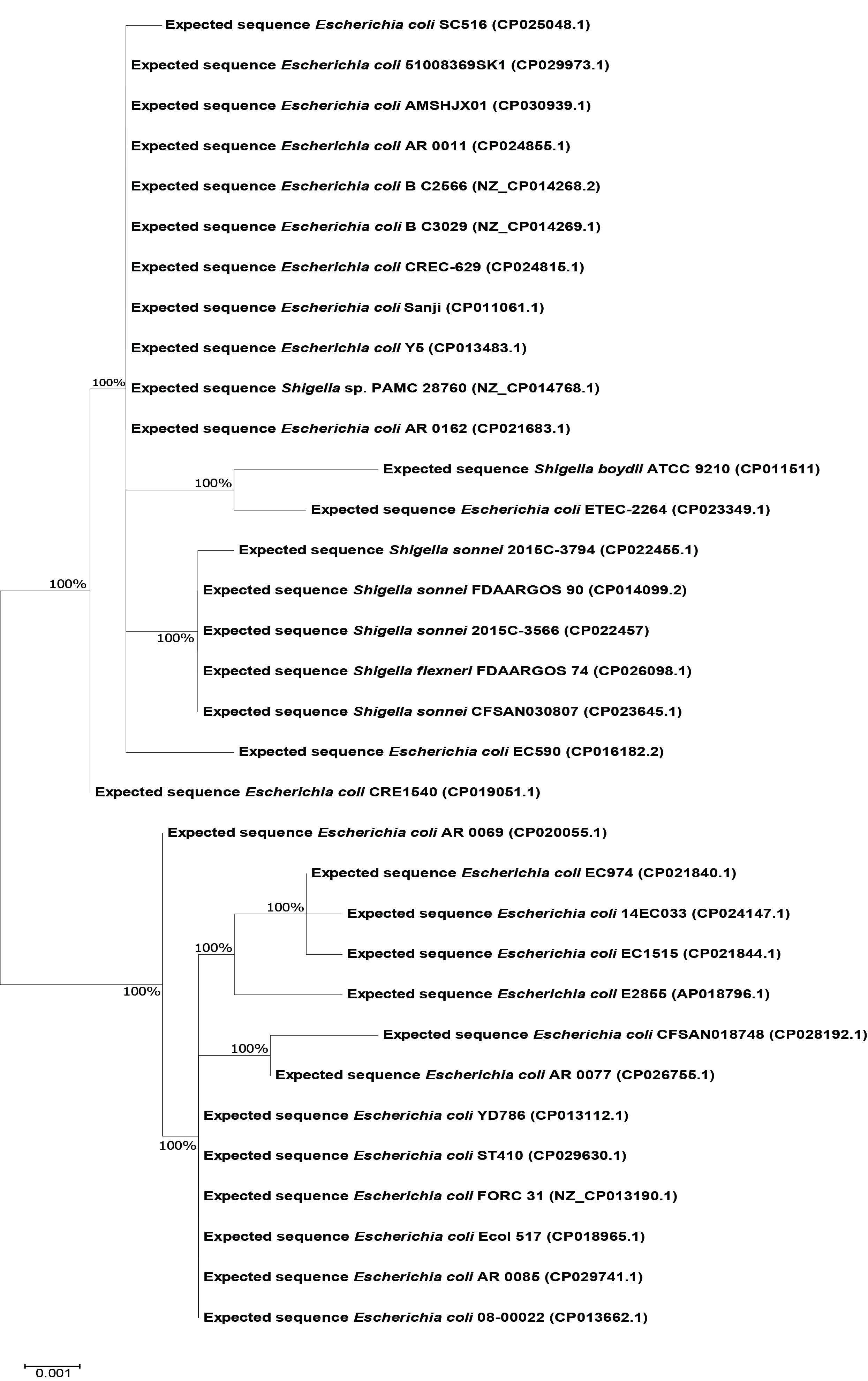

Supplement: S2 Fig — 16S rRNA gene expected sequences from WGS of E. coli strains and Shigella species which are highly similar were compared using a phylogenetic tree. Bootstrap values obtained with 1000 repetitions are indicated as percentages at all branches. The scale bar represents an evolutionary distance of 0.001. GenBank accession numbers of the different E. coli strains and Shigella species are provided in parentheses. (TIF) [file pone.0212090.s002.tif]

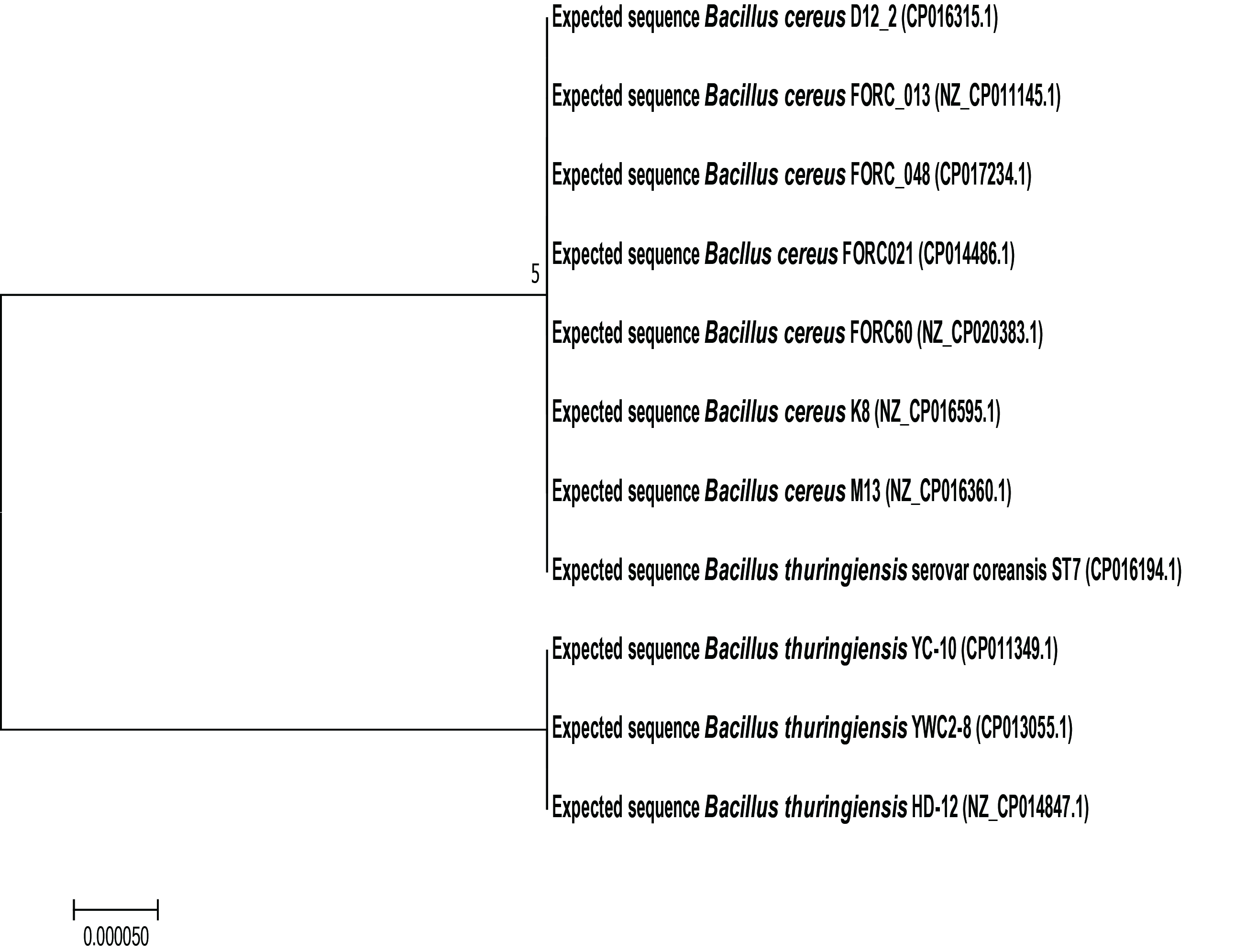

Supplement: S3 Fig — 16S rRNA gene expected sequences from WGS of Bacillus cereus and Bacillus thuringiensis strains which are highly similar were compared using a phylogenetic tree. Bootstrap values obtained with 1000 repetitions are indicated as percentages at all branches. The scale bar represents an evolutionary distance of 0.00005. GenBank accession numbers of the different Bacillus cereus and Bacillus thuringiensis strains are provided in parentheses. (TIF) [file pone.0212090.s003.tif]
