# Supplementary material for: Information about variations in multiple copies of bacterial 16S rRNA genes may aid in species identification
Source: PLoS One. 2019 Feb 15;14(2):e0212090. doi: 10.1371/journal.pone.0212090 (PMC6377111; doi:10.1371/journal.pone.0212090)
Supplement: S1 Table — (DOCX) [file pone.0212090.s004.docx]

**Supplementary Table 1. Representation of the expected values of 7 E. coli strains and 7 Shigella species with the amount of different bases in their respective variation points.**

| *E.coli* 09-0022 |  |  |  |  |  |  |  |  |  |  |  |  |  |  |  |  |  |  |  |  |
| --- | --- | --- | --- | --- | --- | --- | --- | --- | --- | --- | --- | --- | --- | --- | --- | --- | --- | --- | --- | --- |
| Variation points | 81 | 82 | 91 | 92 | 95 | 1004 | 1008 | 1012 | 1021 | 1022 | 1023 | 1024 | 1025 | 1040 |  |  |  |  |  |  |
|  | A/3 | C/6 | G/6 | C/4 | C/6 | A/5 | C/5 | C/5 | G/5 | A/5 | T/5 | T/5 | G/5 | T/5 |  |  |  |  |  |  |
|  | G/4 | A/1 | T/1 | T/3 | T/1 | G/2 | G/2 | T/2 | A/2 | G/2 | A/2 | A/2 | T/2 | C/2 |  |  |  |  |  |  |
| **Expected sequence** | **G** | **C** | **G** | **C** | **C** | **A** | **C** | **C** | **G** | **A** | **T** | **T** | **G** | **T** |  |  |  |  |  |  |
|  |  |  |  |  |  |  |  |  |  |  |  |  |  |  |  |  |  |  |  |  |
| *E.coli* 14EC033 |  |  |  |  |  |  |  |  |  |  |  |  |  |  |  |  |  |  |  |  |
| Variation points | 81 | 82 | 91 | 92 | 95 | 476 | 504 | 1004 | 1008 | 1012 | 1021 | 1022 | 1023 | 1024 | 1025 | 1040 |  |  |  |  |
|  | G/5 | A/5 | T/5 | C/5 | T/5 | A/4 | A/6 | A/6 | C/6 | C/6 | G/6 | A/6 | T/6 | T/6 | G/6 | T/6 |  |  |  |  |
|  | A/2 | C/2 | G/2 | T/2 | C/2 | G/3 | G/1 | G/1 | G/1 | T/1 | A/1 | G/1 | A/1 | A/1 | T/1 | C/1 |  |  |  |  |
| **Expected sequence** | **G** | **A** | **T** | **C** | **T** | **A** | **A** | **A** | **C** | **C** | **G** | **A** | **T** | **T** | **G** | **T** |  |  |  |  |
|  |  |  |  |  |  |  |  |  |  |  |  |  |  |  |  |  |  |  |  |  |
| *E.coli* 51008369SK1 |  |  |  |  |  |  |  |  |  |  |  |  |  |  |  |  |  |  |  |  |
| Variation points | 79 | 80 | 89 | 90 | 93 | 183 | 208 | 226 | 250 | 253 | 273 | 1002 | 1006 | 1010 | 1019 | 1020 | 1021 | 1022 | 1023 | 1038 |
|  | G/4 | C/4 | G/4 | C/4 | C/4 | C/6 | T/6 | G/6 | A/5 | A/5 | T/5 | G/6 | G/6 | T/6 | A/6 | G/6 | A/6 | A/6 | T/6 | C/6 |
|  | A/3 | A/3 | T/3 | T/3 | T/3 | T/1 | C/1 | A/1 | T/2 | T/2 | A/2 | A/1 | C/1 | C/1 | G/1 | A/1 | T/1 | T/1 | G/1 | T/1 |
| **Expected sequence** | **G** | **C** | **G** | **C** | **C** | **C** | **T** | **G** | **A** | **A** | **T** | **G** | **G** | **T** | **A** | **G** | **A** | **A** | **T** | **C** |
|  |  |  |  |  |  |  |  |  |  |  |  |  |  |  |  |  |  |  |  |  |
| *E.coli* AMSHJX01 |  |  |  |  |  |  |  |  |  |  |  |  |  |  |  |  |  |  |  |  |
| Variation points | 80 | 89 | 93 | 98 | 188 | 250 | 253 | 264 | 273 | 1002 | 1006 | 1010 | 1019 | 1020 | 1021 | 1022 | 1023 | 1038 |  |  |
|  | C/5 | G/5 | C/5 | A/6 | C/6 | A/5 | A/5 | C/6 | T/5 | G/4 | G/4 | T/4 | A/4 | G/4 | A/4 | A/4 | T/4 | C/4 |  |  |
|  | A/2 | T/2 | T/2 | G/1 | A/1 | T/2 | T/2 | T/1 | A/2 | A/3 | C/3 | C/3 | G/3 | A/3 | T/3 | T/3 | G/3 | T/3 |  |  |
| **Expected sequence** | **C** | **G** | **C** | **A** | **C** | **A** | **A** | **C** | **T** | **G** | **G** | **T** | **A** | **G** | **A** | **A** | **T** | **C** |  |  |
|  |  |  |  |  |  |  |  |  |  |  |  |  |  |  |  |  |  |  |  |  |
| *E.coli* AR_0011 |  |  |  |  |  |  |  |  |  |  |  |  |  |  |  |  |  |  |  |  |
| Variation points | 81 | 82 | 91 | 92 | 95 | 185 | 210 | 228 | 252 | 255 | 275 | 1004 | 1008 | 1012 | 1021 | 1022 | 1023 | 1024 | 1025 | 1040 |
|  | A/4 | C/4 | G/4 | C/4 | C/4 | C/6 | T/6 | G/6 | A/5 | A/5 | T/5 | G/6 | G/6 | T/6 | A/6 | G/6 | A/6 | A/6 | T/6 | C/6 |
|  | G/3 | A/3 | T/3 | T/3 | T/3 | T/1 | C/1 | A/1 | T/2 | T/2 | A/2 | A/1 | C/1 | C/1 | G/1 | A/1 | T/1 | T/1 | G/1 | T/1 |
|  |  |  |  |  |  |  |  |  |  |  |  |  |  |  |  |  |  |  |  |  |
| **Expected sequence** | **A** | **C** | **G** | **C** | **C** | **C** | **T** | **G** | **A** | **A** | **T** | **G** | **G** | **T** | **A** | **G** | **A** | **A** | **T** | **C** |
|  |  |  |  |  |  |  |  |  |  |  |  |  |  |  |  |  |  |  |  |  |
| *E.coli* AR0069 |  |  |  |  |  |  |  |  |  |  |  |  |  |  |  |  |  |  |  |  |
| Variation points | 81 | 82 | 91 | 92 | 95 | 476 | 692 | 1004 | 1008 | 1012 | 1021 | 1022 | 1023 | 1024 | 1025 | 1040 | 1294 |  |  |  |
|  | G/5 | C/4 | G/4 | C/5 | C/4 | G/6 | G/6 | A/6 | C/6 | C/6 | G/6 | A/6 | T/6 | T/6 | G/6 | T/6 | G/6 |  |  |  |
|  | A/2 | A/3 | T/3 | T/2 | T/3 | A/1 | A/1 | G/1 | G/1 | T/1 | A/1 | G/1 | A/1 | A/1 | T/1 | C/1 | C/1 |  |  |  |
| **Expected sequence** | **G** | **C** | **G** | **C** | **C** | **G** | **G** | **A** | **C** | **C** | **G** | **A** | **T** | **T** | **G** | **T** | **G** |  |  |  |
|  |  |  |  |  |  |  |  |  |  |  |  |  |  |  |  |  |  |  |  |  |
| *E.coli* AR077 |  |  |  |  |  |  |  |  |  |  |  |  |  |  |  |  |  |  |  |  |
| Variation points | 76 | 79 | 90 | 93 | 183 |  |  |  |  |  |  |  |  |  |  |  |  |  |  |  |
|  | G/6 | G/2 | C/2 | C/5 | C/6 |  |  |  |  |  |  |  |  |  |  |  |  |  |  |  |
|  | A/1 | T/3 | A/3 | T/2 | T/1 |  |  |  |  |  |  |  |  |  |  |  |  |  |  |  |
|  |  | A/2 | T/2 |  |  |  |  |  |  |  |  |  |  |  |  |  |  |  |  |  |
| **Expected sequence** | **G** | **T** | **A** | **C** | **C** |  |  |  |  |  |  |  |  |  |  |  |  |  |  |  |

Where the variation points are the position of the variations in the 16S rRNA genes of the genome. The nucleotides present in the points were assessed and the nucleotide that is greater in amount present in the variation point was used to determine the expected sequence.
